# Effluents of Shrimp Farms and Its Influence on the Coastal Ecosystems of Bahía de Kino, Mexico

**DOI:** 10.1155/2013/306370

**Published:** 2013-06-02

**Authors:** Ramón H. Barraza-Guardado, José A. Arreola-Lizárraga, Marco A. López-Torres, Ramón Casillas-Hernández, Anselmo Miranda-Baeza, Francisco Magallón-Barrajas, Cuauhtemoc Ibarra-Gámez

**Affiliations:** ^1^Instituto Tecnológico de Sonora (ITSON), 85000 Ciudad Obregón, SON, Mexico; ^2^Departamento de Investigaciones Científicas y Tecnológicas de la Universidad de Sonora (DICTUS), 83000 Hermosillo, SON, Mexico; ^3^Centro de Investigaciones Biológicas del Noroeste, S.C. (CIBNOR, S.C.), 85454 Guaymas, SON, Mexico; ^4^Universidad Estatal de Sonora (US), 85800 Navojoa, SON, Mexico

## Abstract

The impact on coastal ecosystems of suspended solids, organic matter, and bacteria in shrimp farm effluents is presented. Sites around Bahía de Kino were selected for comparative evaluation. Effluent entering Bahia Kino (1) enters Laguna La Cruz (2). A control site (3) was outside the influence of effluents. Water quality samples were collected every two weeks during the shrimp culture period. Our data show that the material load in shrimp farm effluents changes biogeochemical processes and aquatic health of the coastal ecosystem. Specifically, the suspended solids, particulate organic matter, chlorophyll *a*, viable heterotrophic bacteria, and *Vibrio*-like bacteria in the bay and lagoon were two- to three-fold higher than the control site. This can be mitigated by improvements in the management of aquaculture systems.

## 1. Introduction

Worldwide, brackish-water aquaculture production (4.7 million tons) consisted of crustaceans (57%), freshwater fishes (19%), diadromous fishes (15%), marine fishes (7%), and marine mollusks (2%) in 2010; more than 99 percent of the crustaceans were marine shrimps [1]. It shows the importance of research about the effect of shrimp farming on the environment [2], with water pollution from shrimp pond effluents as the most common complaint [3–5]. This activity depends directly or indirectly on a range of coastal and marine ecosystem services some of which may be used at rates that are not sustainable [6, 7].

Most of shrimp production is carried out in ponds. The most common shrimp aquaculture systems use inland ponds that are near or on the coast. Water is discharged from these shrimp ponds to coastal ecosystem as part of the water exchange when ponds are drained. The main components in the shrimp farm effluents are organic matter mainly in particulate form from different sources, as well as nitrogen and phosphorus in both organic and inorganic forms, and suspended solids [8, 9]. 

Production systems in the culture of marine shrimp, semi-intensive or intensive, lead to significant increases in the levels of nutrients, phytoplankton biomass, organic matter, and suspended solids in the environment receiving the farm's effluents [10–13]. In addition, it has been reported that water quality shows short term increases in parameters of water bodies receiving shrimp discharge waters, but other studies indicate that there are no significant differences over background levels on an annual basis [14, 15]. The impact of pond effluents on adjacent ecosystems is variable and depends on various factors, including the magnitude of the discharge, the chemical composition of the pond effluents, and the specific characteristics of the environment that receives the discharge, such as circulation and dilution rates [16].

The characterization of the shrimp farm's effluents in terms of water quality is very important to gauge the environmental health of an ecosystem in order to achieve a better regulation of the industry [17, 18]. Further, the continuous monitoring of the physical, chemical, and biological parameters of pond, effluent, and inlet waters helps not only to predict and control negative conditions for shrimp farming but also avoids environmental damages and collapse of the production process [19].

In Mexico, about 97% of shrimp aquaculture ponds are located around the Gulf of California in the states of Baja California, Baja California Sur, Sonora, Sinaloa, and Nayarit. Beginning in the mid-1980s, the Gulf of California ecoregion experienced an increase in shrimp aquaculture and became the second largest producer in the western hemisphere [20]. Recently, Mexico was the sixth largest producer of shrimp culture worldwide ~110,000 t [1]. The State of Sonora contributed with 37% of this production in 2011, and specifically Bahía de Kino region which is the main shrimp producer [21].

The goals of this study were to examine the effluent loads from shrimp farms, and its influence on the coast in Bahía de Kino. We measured concentrations of total suspended solids, particulate organic matter, chlorophyll *a*, viable heterotrophic bacteria, and *Vibrio*-like bacteria in the effluent and in the coastal ecosystems. We then assessed the influence of these effluents on the coastal ecosystems.

## 2. Materials and Methods

### 2.1. Study Area

**Figure 1:**
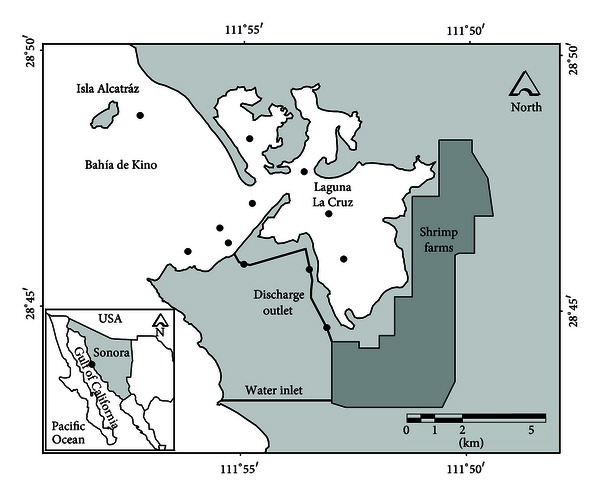
Bahía Kino area, including Laguna La Cruz and Isla de Alcatraz, showing seawater inlet to shrimp farms and discharge channel to the bay.

Bahía de Kino is located on the Gulf of California in the State of Sonora, Mexico (28°47′N, 111°54′W). Into this bay, effluents from 1,350 hectares (effluents rate ~160,000 m^3^ ha^−1^ yr^−1^) from four shrimp farms that operate from April through October are delivered into the bay, about 2 km south of the mouth of the Laguna de la Cruz. Seawater is taken directly from the open sea for shrimp farming (Figure 1). Bahía de Kino has a seasonal pattern of water temperature, a maximum of  32.2°C in August and minimum of 15.6°C in January. Salinity varies from 35–38.3 [22]. This region has a dry desert climate with evaporation of ~3000 mm yr^−1^ which exceeds rainfall of  <300 mm yr^−1^ [23].

### 2.2. Sampling and Measurements

Water quality was sampled every two weeks at 12 sampling stations in June, September, and October (2009), the period when the most effluents and organic matter from partial and final harvest of shrimp takes place.

A control station outside the influence of the shrimp effluents (~6 km) was located at Isla Alcatraz; four stations were established in the southern part of the bay near the outlet of the effluents; three stations were established in the effluent channel; and four stations were established inside Laguna la Cruz (Figure 1).

Water samples were taken between 07:00 and 13:00 h. At each station, water was collected near the surface in 1 L plastic bottles; these samples were used to measure suspended solids, chlorophyll *a*, and pH. Samples for study of bacteria were collected in sterile 120 mL Whirl pack bags. All samples were transported to the laboratory in ice.

At each station, temperature, dissolved oxygen, and salinity were measured (YSI field oxygen meter 85, YSI, Yellow Springs, OH); water transparency was measured with a Secchi disk, and pH was measured in the laboratory with pH meter (model 220A, Hanna Instruments, Woonsocket, RI).

### 2.3. Water Quality Analysis

The water samples collected for the analysis of suspended solids and organic matter were filtered through precombusted and preweighed Whatman GF/C (Whatman International Ltd.) glass fibers. Then, the filters were dried in an oven at 60°C for 24 h and weighed to determine total suspended solids (TSS), for particulate organic matter (POM) estimation (ash-free dry weight), filters and suspended material were then ignited to constant weight at 550°C for 8 h and weighed again. Inorganic suspended solids (ISSs) were estimated by subtracting the value of POM on the TSS [24]. Samples for analysis of chlorophyll *a* were collected by filtration through Whatman GF/C glass fibre filters, extracted with 90% v/v acetone, and measured by spectrophotometry, according to [25].

For the quantification of culturable bacteria, including viable heterotrophic bacteria (VHB), and *Vibrio*-like bacteria (VLB), the spread plate method [26, 27] in Marine Agar 2216 and Thiosulfate-citrate-bile-sucrose agar (TCBS, DIFCO, U.S.A.) was used. A 10-fold dilution series were prepared with 3.0% of sterile seawater diluted with distilled water. The plates were incubated for 48 ± 2 h to 30 ± 2°C, and the bacterial colonies were counted and reported as colony-forming units for mL^−1^ (CFU mL^−1^).

### 2.4. Statistical Data Analysis

We used multivariate, multidimensional, and nonparametric scaling of data which was transformed (log⁡⁡*x* + 1) and standardized to determine whether the sites (island, effluent, bay, and lagoon) were similar in terms of water quality. Statistical software (Primer-E 6.0, Primer-E, Ivybridge, UK) was used to perform the analyses.

The data grouped by area (island, effluent, bay, and lagoon) were subjected to a normality and equal variance test [28]. After this, parametric tests were made through a one way ANOVA with its respective Tukey test of multiple comparisons and also a nonparametric test using one way ANOVA and a Kruskal-Wallis *a posteriori* test with a 95% level of significance [29]. The NCSS program (2007) was used for statistical analysis [30]. 

## 3. Results

**Figure 2:**
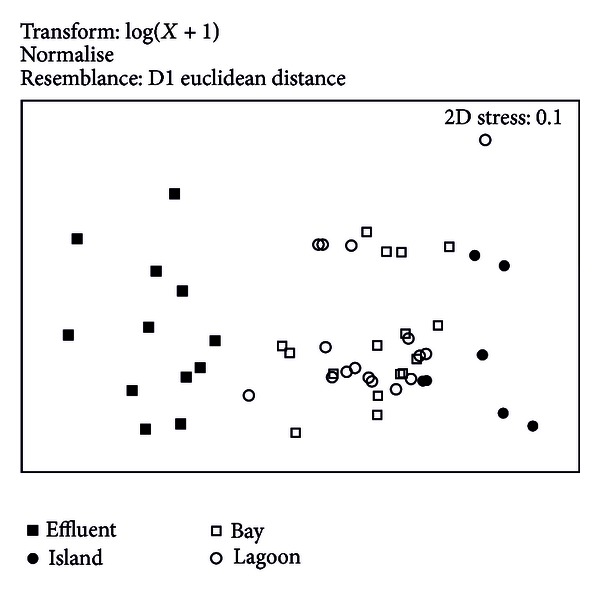
nMDS ordination of water quality parameters for the study areas. Temperature, salinity, dissolved oxygen, suspended solids, particulate organic matter, chlorophyll *a*, *Vibrio-*like bacteria, and viable heterotrophic bacteria were included in the multivariate analysis.

The result from the similarity analysis for the multivariate method of nonparametric multidimensional scaling (nMDS) indicated a difference of similarity among the study areas (Figure 2). Effluents and water quality parameters near the island had lower similarity, while the bay and lagoon had similar water quality conditions, but differed from the water quality conditions of the effluent. 

The average water temperature during all the time of the study on the island was 27.3 ± 3.3°C, effluent of 24.8 ± 5.3°C, at the bay of 25.5 ± 4.6°C, and the lagoon of 26.6 ± 4.4°C, without showing any statistical differences among areas (*H* = 5.27; *P* = 0.15) (Table 1). However, a seasonal behavior was observed when high temperatures were recorded in June and September, and the lowest in October. 

Salinity values were found significantly higher (*F* = 74.5; *P* < 0.001) in the effluent (39.3 ± 1.3) over the island (36.7 ± 0.4), bay (37.2 ± 0.6), and lagoon (37.2 ± 0.4) (Table 1). It was noted that the bay, lagoon, and the control area (island) remained similar in salinities among them.

In the effluent, significantly lower values of DO (4.5 ± 1.3 mg L^−1^) (*F* = 20.2; *P* < 0.001) were found in comparison to the rest of the other study areas (Table 1). No significant differences were found (*P* > 0.05) of DO among the island, bay, and lagoon areas. The pH did not show statistical differences among the areas studied (*H* = 4.41; *P* = 0.22) (Table 1). 

**Figure 3:**
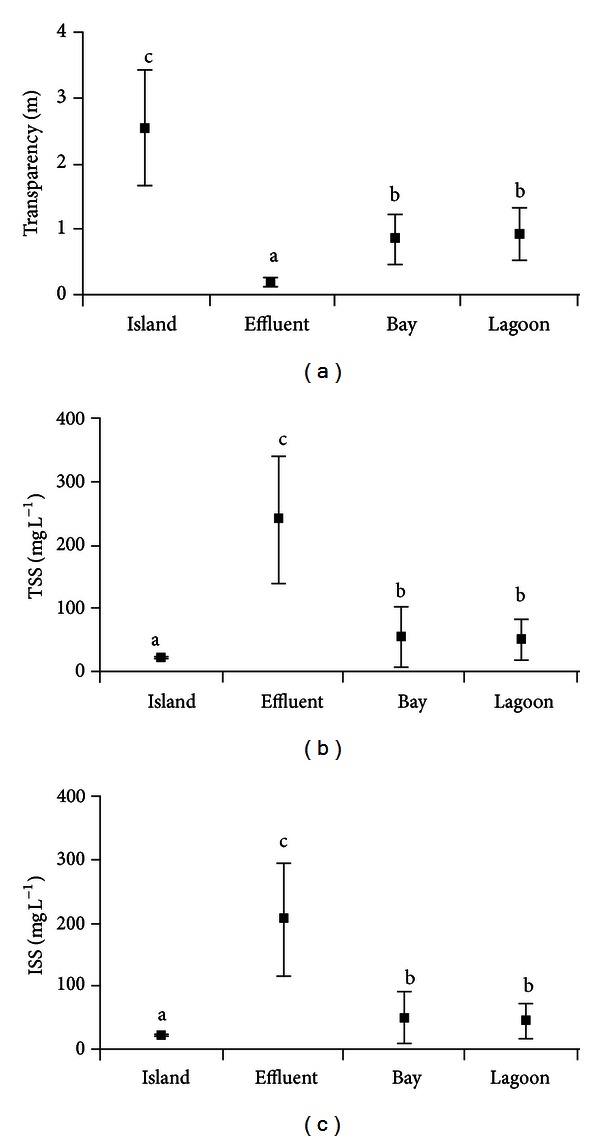
Average values (±SD) of selected variables. (a) Water transparency, (b) total suspended solids, and (c) inorganic suspended solids. Different letters among zones for each variable indicate significant differences (*P* < 0.05).

Water transparency had average values on the island, effluent, bay, and lagoon of 2.5 ± 0.8, 0.2 ± 0.1, 0.9 ± 0.4, and 0.9 ± 0.4 m, respectively (Figure 3). Significantly lower values were recorded in the effluent, and higher on the island (*F* = 34.05; *P* < 0.001). Water transparency both in the bay and lagoon had intermediate values, not statistically different, but significantly lower than the island (Figure 3).

The average concentration of  total suspended solids (TSS) was found to be 26.7 ± 1.2 mg L^−1^ in waters near the island (control area), 233.2 ± 95.7 mg L^−1^ in the effluent area, 56.2 ± 45.1 mg L^−1^ in the bay, and 52.7 ± 30.6 mg L^−1^ in the lagoon. The inorganic suspended solids (ISSs) showed the same pattern as TSS. Both TSS and ISS presented averages significantly (TSS; *H* = 33.15 and *P* < 0.001; ISS: *H* = 33.14 and *P* = 0.001) lower in the island and higher in the effluent, with intermediate values in both bay and lagoon, but significantly higher than near the island (Figure 3).

**Figure 4:**
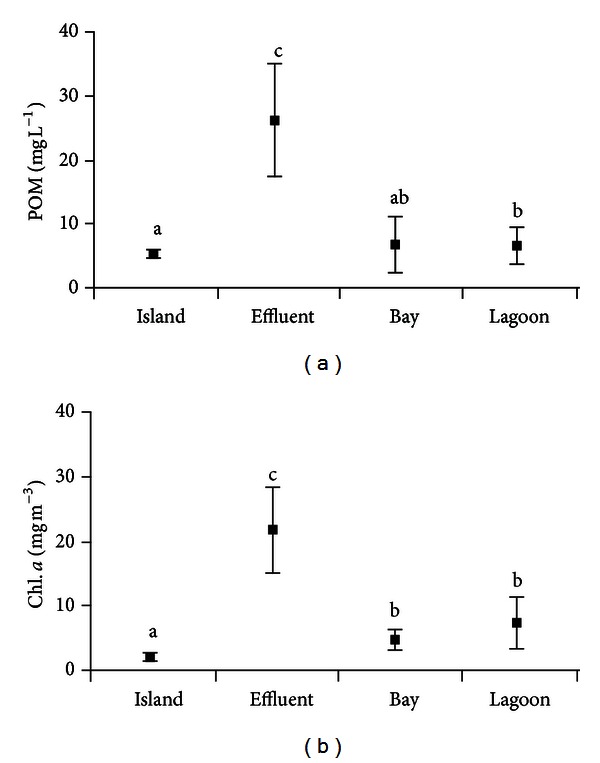
Average values (±SD) of selected variables. (a) Particulate organic matter and (b) chlorophyll *a*. Different letters among zones for each variable indicate significant differences (*P* < 0.05).

Particulate organic matter (POM) had average concentrations near the island of 4.62 ± 0.51 mg L^−1^, 26.1 ± 9.2 mg L^−1^ in effluent zone, 7.1 ±  4.2 mg L^−1^ in the bay, and 7.0 ± 2.7 mg L^−1^ in the lagoon. Significantly higher values (*H* = 31.07; *P* < 0.001) of POM were observed in the effluent zone and lower ones near the island; while POM values in both bay and lagoon remained similar, it was observed that the lagoon presented higher levels than the island (Figure 4).

Chlorophyll *a* concentration in the control area (island) had average values of 2.3 ± 0.7 mg m^−3^, a value which is significantly (*H* = 38.72; *P* < 0.001) lower when it is compared with the other areas (Figure 4). By contrast, significantly higher values (*P* < 0.001) were found in the effluent zone during the complete culture period with 21.9 ± 6.7 mg m^−3^. Chl *a* of bay (4.8 ± 1.4 mg m^−3^) and lagoon (7.5 ± 3.7 mg m^−3^) was maintaining similar values. 

**Figure 5:**
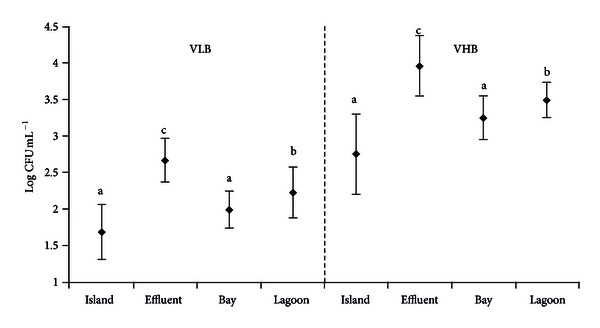
Average concentration (±SD) of *Vibrio-*like bacteria (VLB) and viable heterotrophic bacteria (VHB) in the different areas of study. Different letters among zones for each group of bacteria indicate significant differences (*P* < 0.05).

The average concentration of viable heterotrophic bacteria (VHB) in the control area (island) was of 5.64 × 10^2^ ± 0.35 × 10^1^ CFU  mL−1, while effluent was of 9.07 × 10^3^ ± 0.24 × 10^1^ CFU  mL−1, bay 1.76 × 10^3^ ± 0.20 × 10^1^ CFU mL−1, and lagoon 3.11 × 10^3^ ± 0.18 × 10^1^ CFU mL−1. Significantly higher concentrations (*H* = 45.76; *P* < 0.001) were quantified in the effluent zone and lower ones near the island. There was no difference in VHB between island and bay, whereas concentrations in the lagoon were higher than both island and bay. The *Vibrio*-like bacteria (VLB) presented the same as than the viable heterotrophic bacteria (*H* = 50.6; *P* < 0.001) (Figure 5).

## 4. Discussion

The multivariate analysis (nMDS) showed that water conditions varied considerably among the four areas, especially between the sites in the effluent channel and the other areas. The effluent had no effect on water quality in the control area. The bay and lagoon are similar because there is a large exchange of materials between the two areas, mainly from tidal exchange. Our results suggested that pollutants from the shrimp farm influence environmental conditions in the bay and lagoon.

### 4.1. Water Quality of Shrimp Farm's Effluents

Low dissolved oxygen (DO) in the effluent results from a heavy load of organic matter generated in the shrimp farms [8, 9, 31, 32], which includes unconsumed shrimp food, detritus, phytoplankton, zooplankton, and bacteria. The level of oxygen at the discharge outlet at the bay was 4.5 ± 1.4 mg L^−1^, within the range of water quality standards for shrimp farm effluents recommended by the Global Aquaculture Alliance [33].

Effluent was highly saline because of the high evaporation rates in the shrimp ponds in this subtropical desert region, which is consistent with [34] who report salinity 40–42 in effluents in this region. The suspended solids (TSS and ISS) increased during the course of effluent water flow because the bottom sediments were resuspended and the channel walls were eroded. The effluent was less transparent, caused by the concentrations of suspended solids, POM, and phytoplankton biomass.

Water quality standards for shrimp farm effluents recommended by the Global Aquaculture Alliance for TSS has a standard of  <100 mg L^−1^ and a target standard of  <50 mg L^−1^ [33]. Effluents discharging into Bahía de Kino had TSS concentrations of  233.2 ± 95.7 mg L^−1^.

High phytoplankton biomass and organic matter observed in the wastewater is promoted by inorganic fertilizers in the cultivation system. With current management practices, the ponds are rich in phytoplankton and organic matter, and this water is later discharged [11, 13, 15, 35].

Microorganisms in general and bacteria, in particular, are key elements in the marine ecosystems operation and react quickly to changes in environmental parameters [36, 37]. The quantity of bacteria is an important variable in monitoring of shrimp farm effluent. *Vibrio* bacteria are opportunistic pathogens in shrimp farms at the larval and grow-out stages; it is the most serious pathogen, causing up to 100% mortality [38–40]. 

In summary, our results showed that shrimp farm effluents reaching the bay have higher concentrations of TSS, POM, phytoplankton biomass, and bacteria than the bay; this is consistent with observations in other studies [10–13, 15]. The control site at the island had low concentrations of TSS, POM, phytoplankton biomass, and bacteria, thus, the effluent loadings are still a good indicator of likely impact. The knowledge of those loads is useful for understanding the responses from water bodies receiving shrimp farm effluents.

### 4.2. Influences on Coastal Ecosystems

Sustainability of shrimp culture requires maintenance of good water quality in the adjacent coastal region. Our results showed that the suspended solids, POM, Chl *a*, VHB, and VLB in the bay and lagoon were two- to three-fold higher than the control site. Environmental problems from shrimp farm effluents are associated with water pollution and diseases.

An excess of organic matter discharged into the bay and lagoon induces a higher demand of dissolved oxygen which negatively affects ecosystems by hypoxia. Variations in effluents with low concentrations of dissolved oxygen in the bay and lagoon could be explained by winds pattern [41], tidal mixing, coastal circulation along the coast of the Gulf of California [42], and water exchange time for the lagoon (21 days) [43]. These results suggest that the system was assimilating the organic matter discharged. However, the marginal rate of assimilation by the system does not indicate an absence of ecological impact. High impact may occur during the night in the area surrounding the discharge in the bay, creating hypoxic events, mainly during the summer when winds are less intense, water temperature is higher, and dissolved oxygen is, on average, lower. This represents a potential negative effect on biogeochemical processes and aquatic life.

One of the key environmental concerns about shrimp farming is the discharge of waters with high levels of nutrients into adjacent body waters. Pond water is continuously exchanged (5–30% d−1), with drainage through a ditch that brings back diluted waste water to the bay and inlet channel. With 1,350 ha of shrimp ponds in operation, yielding about 2.5 t ha^−1^ and disposing of 72 kg N and 13 kg P waste for each ton of  harvested shrimp [44], we estimate a load of ~243 t N yr^−1^ and ~44 t P yr^−1^. This contributes to significantly high levels of Chl *a* in the bay, compared to the control site. Nutrient loads have been linked to lower diversity of phytoplankton species and nuisance algae blooms, with impacts the ecological health of coastal ecosystems [45]. In a nearby area in April 2003, a harmful algal bloom of *Chattonella marina*, *C. cf. ovata*, *Gymnodinium catenatum*, and *G*. *sanguineum* caused a massive die-off of fish and mollusks [46]; hence, there is a risk potential of harmful algal blooms.

Most shrimp diseases are bacterial and viral. Most bacterial diseases are caused by *Vibrio* spp. Vibriosis outbreaks is a serious problem in intensive shrimp ponds in this region [47]. These pathogenic bacteria can affect other cultivated marine populations, such as oysters, in this region, as well as species native to the area. When the aquatic environment is enriched by accumulating organic matter, several species of *Vibrio* spp. grow rapidly, not because it has a high growth rate, but because it is adapted to oxygen-deficient conditions [48]. *Vibrio* spp. affects fish, crustaceans, and cultivated shellfish. The important decade-old oyster farming activity (*Crassostrea gigas* and *C*. *corteziensis*) surrounding the bay and lagoon [49, 50] could be impacted by the shrimp farm effluent. These results indicate the need for increased research to determine the consequences of bacterial loads and waste nutrients into the coastal ecosystems.

## 5. Conclusions

Shrimp farm effluent provides significantly high salinity, suspended solids, organic particulate matter, chlorophyll *a*, and bacteria to coastal ecosystems, as well as reduced dissolved oxygen and transparency. Effluent produced changes in water quality in the bay and lagoon. Accumulation of solids, organic matter, and bacterial biomass affects environmental conditions and processes of these ecosystems. There is still insufficient knowledge of how effluents are affecting coastal ecosystems and how it affects aquaculture activities. Current water quality of Bahía de Kino appears to result from inadequate management of shrimp ponds. Our results suggest that both in Mexico and worldwide efforts at waste prevention and minimization at the source and onsite treatment and reuse of effluent would reduce losses of coastal ecosystem services.

## Figures and Tables

**Table 1:** Average values (±SD) of physicochemical parameters for different parts of the study area during discharge of effluent.

Areas	Temperature (°C)	Salinity	Dissolved oxygen (mg L^−1^)	pH
Island	27.3 ± 3.3^a^	36.7 ± 0.4^a^	6.6 ± 1.2^b^	8.3 ± 0.1^a^
Effluent	24.8 ± 5.3^a^	39.3 ± 1.3^b^	4.5 ± 1.4^a^	8.2 ± 0.2^a^
Bay	25.5 ± 4.6^a^	37.2 ± 0.6^a^	5.7 ± 0.9^b^	8.2 ± 0.2^a^
Lagoon	26.6 ± 4.4^a^	37.2 ± 0.4^a^	6.0 ± 0.6^b^	8.2 ± 0.1^a^

Different letters among areas for each parameter indicate significant differences (*P* < 0.05;  *n* = 96).
